# Help-seeking processes related to targeted school-based mental health services: systematic review

**DOI:** 10.1186/s12889-024-18714-4

**Published:** 2024-05-02

**Authors:** Lauren McPhail, Graham Thornicroft, Petra C. Gronholm

**Affiliations:** 1 Centre for Global Mental Health, Health Service and Population Research Department, Institute of Psychiatry, Psychology & Neuroscience, King’s College London, London, UK; 2 Centre for Global Mental Health and Centre for Implementation Science, Health Service and Population Research Department, Institute of Psychiatry, Psychology & Neuroscience, King’s College London, London, UK

**Keywords:** Systematic reviews, School, Mental Health, Adolescent, Intervention, Help-seeking

## Abstract

**Background:**

One in seven adolescents globally are affected by mental health conditions, yet only a minority receive professional help. School-based mental health services have been endorsed as an effective way to increase access to mental health support for people at risk, or currently presenting with mental health conditions, throughout adolescence. Despite this, low treatment utilisation prevails, therefore the aim of this review is to contribute insights into the processes related to adolescents’ accessing and engaging with essential targeted mental health support within schools.

**Methods:**

This systematic review extracted qualitative, quantitative and mixed-methods data to determine what processes affect adolescents seeking help from targeted school-based mental health services (TSMS). Searches were conducted in EMBASE, Medline, PsycINFO, CINAHL, ERIC, Web of Science, in addition to manual searching and expert consultations. Data were synthesised following guidelines for thematic synthesis and narrative style synthesis.

**Results:**

The search resulted in 22 articles reflecting 16 studies with participant sample sizes ranging from *n* = 7 to *n* = 122. Three main themes were identified: ‘access-related factors’, ‘concerns related to stigma’, and ‘the school setting’. These findings elucidate how help-seeking processes are variable and can be facilitated or hindered depending on the circumstance. We identified disparities with certain groups, such as those from low-socio economic or ethnic minority backgrounds, facing more acute challenges in seeking help. Help-seeking behaviours were notably influenced by concerns related to peers; an influence further accentuated by minority groups given the importance of social recognition. Conflicting academic schedules significantly contribute to characterising treatment barriers.

**Conclusions:**

The findings of this review ought to guide the delivery and development of TSMS to facilitate access and promote help-seeking behaviours. Particularly, given the evidence gaps identified in the field, future studies should prioritise investigating TSMS in low- and middle-income settings and through quantitative methodologies.

**Registration:**

The protocol for this systematic review was registered on PROSPERO (ID CRD42023406824).

**Supplementary Information:**

The online version contains supplementary material available at 10.1186/s12889-024-18714-4.

## Background

It is important to understand how adolescents’ access and utilise mental health services, particularly the support provided in school-based settings. Adolescence is a key developmental period when various health issues and mental health concerns can arise [1, 2]. Indeed, approximately 1 in 7 adolescents globally are affected by mental health conditions, with half of all lifetime cases emerging before the age of 14 [2, 3]. During this period, there is a marked increase in disability-adjusted life years (DALYs), with depressive disorders, anxiety disorders and conduct disorders accounting for the largest proportion of DALYs throughout adolescence [4].

Considering the high prevalence of mental health conditions in adolescents, and the possible consequences of suffering and disease burden, many adolescents healthcare needs remain unmet [1]. Adolescents experience barriers in the form of poor mental health literacy, and this lack of knowledge about mental health and services may increase sensitivity to confidentiality, stigma, poor accessibility of care providers, and adverse community attitudes [5–7]. This means fewer than two-thirds of young people with mental health conditions in England receive professional help [5, 8], which can give rise to further problematic consequences, such as adverse pathways to care and worse outcomes throughout the subsequent life course [9, 10]. Despite adolescent service use increasing in some community settings, such as schools, it remains relatively low, with 23.3% of children and young people aged 11 to 16 in England accessing support at school for mental health and wellbeing [11]. It is therefore crucial to understand why low rates of treatment utilisation exist.

Previous studies have identified parent-reported or clinician-reported barriers to accessing services for adolescent mental health [12, 13]. One study reports from the perspective of the school guidance counsellor identifying issues around privacy and confidentiality concerning counselling service provision, and underuse of services, even with evident need amongst students [14]. There has been a notable growth of research conducted to explore adolescents’ perspectives on perceived barriers and facilitators to accessing formal sources of help from mental health services [15–17]. However, the extent of this research, as reported by adolescents in school settings, is yet to be investigated [18].

Schools have historically been recognised as one of the largest providers of mental health services for young people, and more recently, have been endorsed by the WHO as an effective place to tackle health conditions in adolescence [6, 19]. Schools are especially significant for their role in providing equitable access to adolescents from diverse backgrounds, considering that they spend many of their waking hours in these settings [20]. Given their well-established position in delivering mental health care [21], there has been a notable effort to increase the availability of services within this setting. In England, for example, the government has increased funding to more than £17 million between 2021 and 2025 and begun to rollout policies such as Mental Health Support Teams in schools, to improve mental health and well-being support in schools and colleges [22, 23].

Despite this, a dichotomy exists where, on one hand, it is argued that schools provide a familiar and non-stigmatising service setting increasing access for those who might otherwise not seek help [24]. On the other hand, a systematic review by Gronholm and colleagues countered this by suggesting that targeted school-based mental health services risk stigmatising adolescents who access them [25]. They conclude the widespread and pervasive effects of stigma may limit the increased access to mental health services through targeted school-based provision. Similar findings report on stigma as one of the most prominent barriers to mental health help-seeking for common mental health conditions among adolescents and also among older populations more generally [9, 26].

Most commonly, school-based mental health services take either a targeted or universal approach. Targeted services, whether selective or indicated, are offered in an individualised or group format [27]. When comparing the two approaches, ‘selective’ aim to address mental health concerns among those at risk, whilst ‘indicated’ target individuals with more pronounced mental health needs, focusing on treatment [27]. In contrast, universal services take a whole-school, generalised approach, regardless of individual risk or need, and have the potential to prevent mental health conditions in larger groups [27]. Yet there is little consensus on which is best suited to address adolescent mental health concerns [28]. Specific worries have been raised on the potential iatrogenic effects of universal services, whereby some students might be taught unhelpful or irrelevant information that may actively cause harm. This could be due to the generalised and widespread dissemination of information, where individual concerns are unknown, thus potentially increasing the risk of internalising symptoms [24]. Targeted services, however, hold promise to be more effective in tailoring care [29]. This has been noted during the childhood-adolescent transition as a crucial period in which targeted support for mental health should be provided [3].

Previous reviews have largely focused on synthesising universal and/or targeted school-based mental health services to establish their effectiveness in preventing or treating adolescent mental health [30–34]. To date, only one systematic review synthesises studies reporting on accessing and utilising TSMS, however, this is limited to help-seeking influences in terms of stigma, qualitative data, and includes multiple informants rather than a focus on the adolescents’ perspectives [25]. In this context, the purpose of this systematic review is to enhance our understanding of help-seeking processes related to TSMS. One approach to consider help-seeking processes is by examining the interplay between different factors, such as facilitators and barriers, associated with help-seeking. Recognising how these factors interact is crucial to effectively promote help-seeking behaviours and to address the diverse needs of adolescents with mental health concerns. The intended value of this review may therefore have implications for future research and practice by identifying key evidence gaps and ways to overcome treatment barriers within the school setting, and finally, informing intervention design for TSMS.

### Study aims and objectives

The aim of this systematic review is to investigate what help-seeking processes affect adolescents accessing and utilising TSMS. We intend to address the overall research question: “What processes affect adolescents seeking help from TSMS?” through addressing the following objectives: (i) To synthesise adolescents’ reported help-seeking processes in relation to accessing and utilising TSMS; and (ii) To contrast any subgroup differences based on evidence coming from high-income settings (HIS) and low-middle-income settings (LMIS).

## Methods

This review adheres to the 2020 Preferred Reporting Items for Systematic Reviews and Meta-Analyses (PRISMA) statement (see Supplement 1) [35]. The protocol for this systematic review was registered on PROSPERO (ID CRD42023406824).

### Search strategy

The search strategy was developed in EMBASE, guided by the aforementioned systematic review on stigma related to TSMS, and subsequently translated to 5 electronic databases – MEDLINE, PsycINFO, CINAHL, ERIC and Web of Science [25]. Each search included MeSH terms and keywords related to the following topics: (barriers or facilitators or help-seeking) AND (adolescents) AND (mental health) AND (school-based interventions or services) (see Supplement 2 for the full search strategy). In addition to the database search, we performed manual hand searching by forward and backward citation tracking of all included papers and related systematic reviews. Content experts (authors of articles identified for inclusion) were contacted for further paper recommendations.

### Inclusion and exclusion criteria

The review eligibility criteria are outlined in Table 1. Included studies investigated TSMS containing qualitative, quantitative, and/or mixed-methods data on help-seeking processes, reported by adolescents themselves, reflecting their experiences related to accessing and utilising these services. Studies focused on help-seeking processes related to other services (i.e., outpatient clinics) were excluded. This was to ensure services could be comparable and prevent significant heterogeneity, since services outside of schools typically involve a different care setting. TSMS, with the primary aim of supporting adolescents’ mental health, of any form (i.e., in person, group or online), were eligible for inclusion. Studies including both those who had received treatment and those who had not, or comprised different informants were included if the findings could be separated; this was mainly relevant for quantitative studies where the overall effect between both groups were analysed together. Studies that were both universal and targeted were excluded, as well as studies that were both predominantly school-based and home-based, or existed in another setting due to their distinct approaches that govern different help-seeking processes.

**Table 1:** Inclusion and exclusion criteria

Help-seeking Processes	
**Inclusion criteria**	**Exclusion criteria**
[1] Studies reporting on adolescent help-seeking processes (barriers and facilitators, help-seeking attitudes, intentions, and behaviours) that focus on seeking, utilising and engaging with formal sources of care (i.e., school counsellor, school psychologist)	[1] Studies reporting on such processes concerning informal sources of help (i.e., family and peers) and self-help
***School-going Adolescents***	
**Inclusion criteria**	**Exclusion criteria**
[1] Adolescents attending school-level education [2] identified as having mental health difficulties, or being at risk of such difficulties, by meeting screening criteria for and/or participating in TSMIs (i.e., depression, anxiety)	[1] Adolescents neither screened for nor participated in TSMIs [2] adolescents’ primary health condition is not mental health (i.e., autism spectrum disorder (ASD), substance use disorder)
***Adolescent Mental Health***	
**Inclusion criteria**	**Exclusion criteria**
[1] Measures of mental health, including but not limited to mental health and well-being; pre-clinical psychological conditions or mental health conditions measured by a validated/commonly used rating scale, or by a structured psychiatric diagnostic interview [2] measures of other individual-level domains related to mental health (i.e., cognitive function, self-concept, emotional regulation, coping skills) [3] identified as at risk (i.e., by teacher/parent referral, self-referral)	[1] Mental health is not the primary outcome [2] mental health and another non-mental health-related co-occurring disorder/disability (i.e., anxiety and ASD)
***School-based Targeted Mental Health Interventions***	
**Inclusion criteria**	**Exclusion criteria**
[1] Targeted approach delivered by Tier 2 (selected) or Tier 3 (indicated) programs, as defined in the Multi-Tiered System of Supports (MTSS*) framework [2] interventions with the primary aim of supporting adolescents’ mental health, (i.e., psychotherapeutic interventions, social and emotional learning interventions) [3] conducted in an individual or group setting [4] provided in a school-based setting (in-person or online) [5] provided in school-settings below university level	[1] Interventions with a universal approach (i.e., whole-school interventions, interventions without targeting a specific risk/symptom) [2] interventions with the primary objective to support adolescents’ non-mental health-related issues (i.e., learning difficulties, physical health conditions, alcohol/drug use) [3] Interventions outside the school setting (i.e., home-based, residential institutions, juvenile placements) [4] interventions targeting and/or involving both adolescents and caregivers (i.e., parents) [5] interventions that are both targeted and universal
***Methodology and Study Type***	
**Inclusion criteria**	**Exclusion criteria**
[1] Studies utilising qualitative, quantitative, or mixed-method design [2] data-based/primary studies [3] studies published in peer-reviewed journals [4] published in English language [5] full-text studies available	[1] Qualitative, quantitative or mixed-methods studies not addressing this review’s research question [2] non-data-based/secondary studies; (i.e., reviews, meta-analyses or meta-syntheses, editorials, protocols, commentaries, letters) [3] non-peer-reviewed work (i.e., conference abstracts, theses, grey literature) [4] studies published in another language than English [5] studies for which full-text cannot be accessed

*MTSS (Arora PG, Collins TA, Dart EH, Hernández S, Fetterman H, Doll B. Multi-tiered Systems of Support for School-Based Mental Health: A Systematic Review of Depression Interventions. School Mental Health. 2019 Jun;11 [2]:240–64)

A broad approach to mental health was taken with no limit to the mental health condition being studied. Hence, school-going adolescents identified as having mental difficulties, or being at risk of such difficulties were included. Adolescents were defined as young people aged 10 years to 19 years [2]. Services including parents were excluded, as they may cross the boundary of existing outside the school-setting; therefore, by excluding services that involve parents, this review can better isolate the processes relevant to the adolescent and school-setting. In this review, studies were considered to assess help-seeking processes related to TSMS if they explicitly used quantitative measures such as, the Barriers to Treatment Participation Session questionnaire [36], self-reported by the adolescent; or qualitative reflections on accessing or utilising TSMS, for example, as prompted in semi-structured interviews. Studies reporting on acceptability, satisfaction or attendance of the intervention were deemed insufficient to report on help-seeking processes. No restrictions were applied on target populations, country setting or publication date.

### Study selection

Electronic database searching took place in March and April 2023 to identify articles relevant for inclusion per the eligibility criteria (Table 1). All records were exported to reference management software EndNote 20 for de-duplication and following this, the records were exported to Rayyan for screening. We screened the initial search results for relevance based on titles and abstracts, with 15% of the total records screened by two reviewers separately. To ensure both reviewers jointly understood the eligibility criteria, a subset of the total records (*n* = 200) were initially independently screened by the two reviewers, with any conflicts resolved through discussions. This was then repeated once all records for independent screening by the two raters were complete. LM independently screened the remaining and the second rater was engaged for resolution when necessary. Full-text versions of studies deemed potentially relevant were retrieved and screened against inclusion criteria. The second reviewer independently screened 15% of the total full-text papers. An initial subset (*n* = 15) of the full-text papers were screened to establish screening consistency and any disagreements were discussed and resolved. After we completed screening all full-texts, any remaining conflicts were resolved through collaborative discussions. All full-texts were available online, therefore we did not need to contact study authors at this stage.

### Data extraction

Using a standardised, pre-piloted form designed in Excel, data were extracted on study design (qualitative, quantitative, mixed-methods); study setting (country, school setting); study aims/objectives; intervention characteristics (description, delivery method); participant data (age, gender, ethnicity, mental health characteristics); and self-reported data on help-seeking processes (attitudes, behaviours, barriers, or facilitators) in relation to intervention participation. The data extraction process was conducted by the first author. Qualitative data were extracted mostly from participant utterances (i.e., interviews and focus groups) as reported in the included articles on the topic of help-seeking processes related to TSMS, while content from author’s results narrative and discussion were extracted too. Relevant quantitative data (i.e., survey data on TSMS access predictors) were also extracted.

### Quality assessment

The Mixed Methods Appraisal Tool (MMAT) was employed to assess the methodological quality of included articles [37]. The MMAT consists of two generic screening questions (a) clarity of research question(s), and (b) appropriateness of the data for addressing the research questions, and for quantitative and qualitative studies a further five methodology-specific questions [37]. For mixed-methods studies, there are three sets of five methodology-specific questions (qualitive, quantitative, and mixed methods), resulting in a total of 15 quality questions [37]. One point was awarded for meeting the criteria for a given question. For partially met criteria, half a point was awarded. Where the criteria was not met, or it was not possible to assess whether the criteria was met due to insufficient level of information provided in the article, no point was awarded [25]. For quantitative and qualitative studies, the MMAT score ranged from 0 to 5 and mixed-methods studies 0–15. An overall percentage was calculated from the total MMAT score (dividing an article’s MMAT score with the maximum total score) which determined the quality level of each included study, with 0% indicating low quality and 100% high quality. Included studies were assessed for quality by the first author. Studies were not excluded based on methodological quality.

### Data synthesis

The main synthesis addressing review objective (i) To synthesise adolescents reported help-seeking processes in relation to accessing and utilising TSMS, follows scholarly guidance on conducting a narrative synthesis and thematic synthesis [38, 39]. Due to expected methodological variation and heterogeneity in the included studies, owing to the broad nature of this review, a quantitative synthesis was not planned. Rather, a narrative-style synthesis was intended to combine findings from included quantitative studies [38]. To combine qualitative data we thematically synthesised findings following the steps to conduct a thematic synthesis [39]. This process involved extracting contextual data from the articles and deriving codes that develop into iterative key themes and subthemes. We then triangulated qualitative and quantitative data to discuss the findings together on help-seeking processes related to TSMS in the synthesis. The was led by the first author and collaborative discussions with the co-authors confirmed accuracy. To address the secondary review objective (ii) To contrast any subgroup differences based on evidence coming from HIS and LMIS, if feasible, we planned to compare study results by their setting (HIS vs. LMISs) to identify patterns, convergences, or divergences within these data.

## Results

**Fig. 1:**
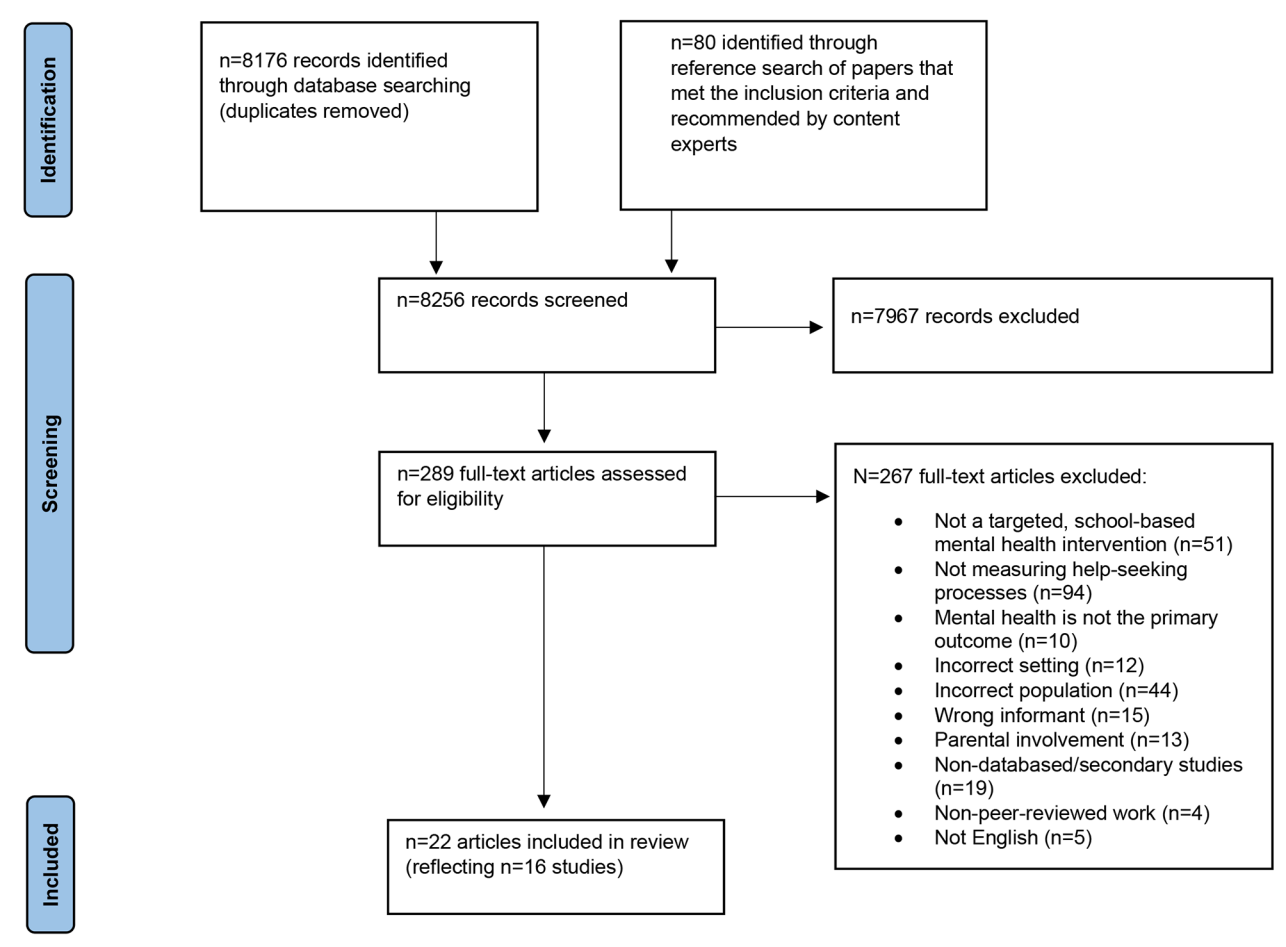
PRISMA flow diagram summarising the article selection process

A total of 8176 non-duplicated records were retrieved from the database search; 7967 of these were excluded after title and abstract screening. We accessed full-texts for the remaining 209 records of which 200 were excluded. We identified an additional 80 articles via manual hand searching, of which 13 were included in the review upon meeting inclusion criteria. In total, 289 full-text articles were reviewed, and 22 articles met the criteria for inclusion. For the content expert consultation, we contacted the corresponding authors of each included study, of whom 6 replied. Some provided additional articles unidentified by the initial search, but none met the criteria for inclusion. The article selection process is depicted in Fig. 1.

The 22 articles included in this review represent 16 studies, with an aggregate total participant sample size of *n* = 532 – children and adolescents who participated in the service.

The main characteristics of the included studies are reported in Tables 2 and 3. Most included articles described a qualitative design (*n* = 20; 90%); only one quantitative (5%) and one mixed-methods article (5%) were included. Only the qualitative data presented in the mixed-methods article were relevant for this review, as the quantitative data did not measure help-seeking processes. In terms of school setting, for most of the included articles this was described as secondary or high school level (*n* = 11; 50%) [40–50]. Some articles (*n* = 9; 41%) did not specify the school level but reported the age of participants – for example 12–18 years – therefore, by association it was presumed this was secondary-level schooling [18, 51–58]. Only one article considered primary school level (*n* = 1; 5%) [59]. Another article did not specify the school level but provided an age range of students between 6 and 18 years (mean 11.03 years), this was ultimately presumed to consider both primary and secondary level schooling (*n* = 1; 5%) [36]. Regarding participant characteristics, most included articles had an age range within the prespecified adolescence period between the age of 10–19 years (*n* = 16; 73%) [1, 18, 40–43, 46–50, 53–58]. The age range of the remaining articles did not fully align with the specified range, however, of these, five articles (23%) provided a mean age value within this range and one article (5%) participants’ ages ranged from 9 to 12 years, which was considered satisfactory for their inclusion [36, 44, 45, 51, 52, 59]. With the ages of some participants below the adolescence age range, as defined by the WHO as between 10 and 19, we refer to participants as children and young people (CYP) in the results and discussion herein. Participants’ ethnicity and race when reported was varying. Through their involvement in the services described in the included studies, adolescents were identified as having mental difficulties, or being at risk of such difficulties. Most services utilised a screening criterion or a referral process (*n* = 14; 64%) [18, 36, 40–43, 46, 49–52, 55, 58, 59]. Two interventions (9%) were offered universally in a targeted manner to a group of adolescents deemed ‘at risk’ [53, 54]. The remaining articles did not specify the service criteria – however, it can be presumed that the students self-referred or were referred due to some perceived risk (*n* = 6; 27%) [44, 45, 47, 48, 56, 57].

**Table 2:** Study characteristics

Study Identification	Study Aims	Study Design	Country	School Setting	*n adolescents (n total sample)*	Gender	Age	Ethnicity
Dickinson et al. (2003)	To determine whether the TRAVELLERS programme was an appropriate, feasible, acceptable, and promising intervention for young people	Mixed-methods (only qualitative data used for this review)	Aotearoa, New Zealand	*n* = 2 secondary schools (one rural and one urban)	*n* = 34	Females *n* = 24 Males *n* = 10	13–14 years (mean not reported)	Mainly Pakeha/European
Evans et al. (2015)	To explore young people’s lived experiences of participating in a targeted SEL intervention; to generate new theoretical and empirical insights into the manifestation of iatrogenic effects within the educational domain	Qualitative	Wales (UK)	*n* = 4 secondary schools (*n* = 3 in post-industrial towns, *n* = 1 in a rural area)	*n* = 41	Females 50% Males 50%	12–14 years (mean not reported)	“White background”
Fazel (2015)	To explore the role of schools in supporting the overall development of refugee and asylum-seeking children in the UK	Qualitative	Glasgow, Cardiff and Oxford (UK)	*n* = 3 schools located in Glasgow, Cardiff and Oxford (*n* = 1 per location)	*n* = 40	Females *n* = 11 Males *n* = 29	15–24 years (mean age 17 years)	not reported
Fazel et al. (2016)	To understand the experience of adolescents directly seen by school-based mental health services; concerning their experience of being seen within the school location, how they perceived therapy, whether it helped them, and finally, any worries that might be impacting their time at school	Qualitative	as above	as above	as above	as above	as above	Albania [5]; Somalia [4]; Sudan [3]; Iran/Iraq ‘Afghanistan [9]; other Asia [7]; South America [2]
Gampetro et al. (2012)	To explore the perceptions of mental health needs of 18 inner-city teens diagnosed with behavioural or mental health issues	Qualitative	Chicago (US)	*n* = 1 school (low resource community)	*n* = 18	Females *n* = 10 Males *n* = 8	12–18 years (mean not reported)	African American [12]; Hispanic [5]; Native American [1]; Caucasian [1]
Garmy et al. (2015)	To explore adolescents’ experiences with a school-based cognitive-behavioural depression prevention program	Qualitative	Sweden	*n* = 6 schools (in four municipalities in rural and urban areas of southern Sweden)	*n* = 89	Female 75% Male 25%	13–15 years (mean not reported)	not reported
Gibson et al. (2013)	To find out how young clients made sense of their experiences of counselling and whether this would be similar or different to the way that the professional literature constructs counselling	Qualitative	New Zealand	*n* = 2 high schools	*n* = 22	Females *n* = 15 Males *n* = 7	16–18 years (mean not reported)	New Zealanders of European ancestry [11], Maori or Pasifika [5], ‘immigrants from other English-speaking countries’ [6]
Gibson et al. (2014)	To explore how young clients who made use of a school counselling service understood their counselling experience	Qualitative	as above	as above	as above	as above	as above	as above
Harrison (2019)	To investigate the processes by which Hong Kong Chinese secondary school students engage with school counselling services from the perspective of both clients and counsellors, and how the sociocultural context and the school setting influence these processes	Qualitative	Hong Kong, China	*n* = 3 secondary schools (private and coeducational)	*n* = 25 (total sample *n* = 33)	Females *n* = 16 Males *n* = 9	14 years+ (mean age 16.7 years)	Chinese (local Hong Kong)
Harrison (2022)	To research the change processes experienced by adolescent Hong Kong Chinese clients, considering the voices of service users and providers	Qualitative	as above	as above	as above	as above	as above	as above
Kendal et al. (2011)	To evaluate The Project’s feasibility and acceptability from the perspectives of staff and students in those schools	Qualitative	England (UK)	*n* = 3 high schools (located in socio-economically deprived urban areas of Northern England)	*n* = 9 (total sample *n* = 50)	Not specified for each informant group	11–18 years	not reported
Kit et al. (2019)	To explore Singapore Asian primary school children’s experiences of online live chat counselling, to gain insight into their motivations for engaging in help-seeking behaviours, and the utility of providing online counselling services to this population	Qualitative	Singapore	*n* = 1 primary school	*n* = 23	Females *n* = 15 Males *n* = 18	9–12 years (mean not reported)	Ethnic mix of Chinese (*n* = 17), Malays (*n* = 9), Indians (*n* = 6) and another race (*n* = 1)
Kvist Lindholm & Zetterqvist Nelson (2014)	To explore how the programme is constructed through the girls’ descriptions and arguments, which contradicts the official version of DISA	Qualitative	Sweden	*n* = 6 schools (located in a relatively small municipality in Sweden)	*n* = 32	All female	12–14 years (no mean reported)	not reported
McKeague et al. (2018)	To investigate the feasibility of the DISCOVER workshop programme for students in UK sixth forms	Qualitative component of a cluster RCT	London, England (UK)	*n* = 10 schools (inner economically deprived and ethnically diverse city area)	*n* = 15 (total sample *n* = 34)	Females *n* = 12 Males *n* = 3	16–19 years (mean age 17.59 years)	Black British, African [6]; Black British, Caribbean [3]; White British [4]; other BME group [2]
Nabors et al. (1999)	To investigate student perceptions about program strengths and weaknesses; barriers to participating in treatment; and treatment outcomes. In particular, the study aims to examine gender differences in response to focus group questions about mental health services for adolescents.	Qualitative	Baltimore City, Connecticut (US)	*n* = 3 high schools (inner city area)	*n* = 37	Females *n* = 24 Males *n* = 13	14–19 years (mean age 16.4 years)	African American [30]; Caucasian [7]
Nabors et al. (2000)	To explore stakeholder perceptions on the strengths, weaknesses, and outcomes of the ESMH program	Qualitative	as above	as above	*n* = 37 (total sample *n* = 108)	as above	as above	as above
Pella et al. (2018)	To examine anxious children’s perceptions of barriers to treatment attendance in a school-based setting and their association with demographic factors, child, and parent clinical characteristics, parenting style and parent service use history	Quantitative (RCT)	Baltimore City, Connecticut (US)	Not reported	*n* = 122	Female 51.6% Male 48.4%	6–18 years (mean age 11.03 years)	Non-white (50.9%); Asian (2.7%); African-American (35.7%); Hispanic (8%); more than one race (4.5%)
Prior (2012a)	To analyse young people’s narratives of accessing a school counselling service	Qualitative	Central Scotland (UK)	*n* = 1 school (located in central Scotland)	*n* = 8	Females *n* = 6 Males *n* = 2	13–17 years (mean not reported)	Not reported
Prior (2012b)	To elucidate the key features and stages of the help-seeking process as defined by young people accessing school counselling	Qualitative	as above	as above	as above	as above	as above	as above
Segrott et al. (2013)	To establish Bounceback’s aims, feasibility and acceptability, through: [1] exploring the view of young people who used the service in relation to acceptability and perceived outcomes; [2] examining Bounceback’s potential to prevent emotional/mental health issues in young people in becoming more severe; [3] examining the relationship between Bounceback and schools in which it operated; [4] identifying young people’s support needs during the transition from school to adulthood	Qualitative	Wales (UK)	*n* = 3 schools (located in south Wales, serving economically disadvantaged city populations and pupils from ethnic minority backgrounds)	*n* = 7 (total sample *n* = 16)	Females *n* = 3 Males *n* = 4	14–16 years (mean not reported)	not reported
Van de Water et al. (2018a)	To compare the experiences and perceived efficacy of two PTSD interventions by treatment users (adolescents with PTSD) and treatment providers	Qualitative study nested within the main RCT study	Cape Town, South Africa	*n* = 4 high schools (lower income)	*n* = 10 (total sample *n* = 13)	Females *n* = 8 Males *n* = 2	13–18 years (mean not reported)	not reported
Van de Water et al. (2018b)	To report on the experiences of stigma in adolescents participating in the RCT and use this knowledge to inform the wider implementation of these interventions for PTSD, especially in LMIS’s	as above	as above	as above	*n* = 10	as above	as above	not reported

**Table 3:** Intervention characteristics

Study Identification	Intervention	Delivery Method	Participants Mental Health	Key Themes Related to Help-seeking Processes
Dickinson et al. (2003)	TRAVELLERS: a school-based early intervention programme helping young people manage and process change, loss and transition	Group-based; in-person	Screening procedure (operates as a filter from the first to the last): answering no to self-report measure “do you feel good about yourself most of the time?“; or those who scored 40 and above on the Subjective Experience of Distress scale; or those who rated four of more life-events with major impact; or those who had attended 7 or more schools were also included	Anticipated stigma; navigating through stigma
Evans et al. (2015)	The Student Assistance Programme (SAP): a targeted school-based SEL intervention	group-based; in-person	Students experiencing social and emotional problems, particularly within school and the family. The SAP stipulates four referral routes for the intervention: self-referral; teacher referral; parental referral; community services referral	Anticipated stigma; negative labelling; navigating through stigma
Fazel (2015)	School-based mental health services for refugee children	individual, group or multimodal; in person	Refugees or asylum seekers identified as at risk and referred by teachers	Referral agent; confidentiality and trust; negative labelling; anticipated stigma
Fazel et al. (2016)	as above	as above	as above	Available and accessible care; anticipated stigma; referral agent; confidentiality and trust; navigating through stigma
Gampetro et al. (2012)	School-based health clinic	individual; in person	Adolescents with an Axis 1 diagnosis who were seen by the school-based health clinic’s licensed clinical social worker (LCSW). The adolescent also had to have received a DSM-IV-TR diagnosis during the one-year period data were being collected	Confidentiality and trust; available and accessible care
Garmy et al. (2015)	DISA (‘depression in Swedish adolescents’): a school-based cognitive-behavioural depression prevention program.	Group-based; in-person	Adolescents deemed at risk due to their age between 13–15 years olds. This age group was chosen because adolescents at this age are considered sufficiently mature to grasp the theory of the program and because depression rates in this age group have been increasing	Anticipated stigma; negative labelling; navigating through stigma
Gibson et al. (2013)	School counselling service	individual; in person	Students can self-refer or can be referred to counselling by a friend, teacher, or other professional.	Individual agency; referral agent
Gibson et al. (2014)	as above	as above	as above	A direct solution to a problem; misconceptions of the service
Harrison (2019)	School counselling service	individual; in person	Not specified	Available and accessible care; misconceptions of the service; confidentiality and trust; negative labelling
Harrison (2022)	as above	as above	Not specified	Available and accessible care; confidentiality and trust
Kendal et al. (2011)	“The Change Project” (The Project): an intervention to promote EWB in high schools	individual; in person	Students self-referred for emotional difficulties including anxiety, low mood, self-esteem, and relationship problems	Individual agency; referral agent; available and accessible care; competing academic schedules
Kit et al. (2019)	Online counselling portal, part of the iZ Hero Challenge	individual; online *(within scheduled after school hours)*	Students experiencing socio-emotional distress were identified by teachers through the school’s participation in the iZ Hero Challenge. They identified 228 nine- to 12-year-old children in need of help. *Through teachers referrals a* total of 33 children (18 males, 15 females) aged between 9 to 12 years old agreed to participate	Direct solution to a problem; confidentiality and trust; available and accessible care
Kvist Lindholm & Zetterqvist Nelson (2014)	DISA (‘depression in Swedish adolescents’): a school-based cognitive-behavioural depression prevention program.	Group-based; in-person	Girls at risk of developing depression. Offered to girls specifically; to address concerns about teenage girls’ mental health and is based on the view that this group is ‘at risk’ for developing depression	Anticipated stigma; negative labelling
McKeague et al. (2018)	DISCOVER: “How to handle stress” workshop programme. This is a self-referral school-based group intervention designed for stressed students in sixth form	group-based; in person	Students self-referred owing to self-perceived need for psychological support in managing common adolescent stressors	Available and accessible care; competing academic schedules; confidentiality and trust; navigating through stigma
Nabors et al. (1999)	Expanded School Mental Health Program	individual; in person	Not specified	Competing academic schedules; negative labelling; confidentiality and trust; available and accessible care
Nabors et al. (2000)	as above	as above	Not specified	Anticipated stigma; competing academic schedules; referral agent
Pella et al. (2018)	STARS: School-based treatment for anxiety research study	individual; in person	DSM-IV primary diagnosis of an anxiety disorder based on the Anxiety Disorders Interview Schedule for DSM-IV	Competing academic schedules; confidentiality and trust; anticipated stigma
Prior (2012a)	School counselling service	individual; in person	Not specified	Anticipated stigma; negative labelling
Prior (2012b)	as above	as above	as above	Anticipated stigma; referral agent; confidentiality and trust; individual agency; direct solution to a problem
Segrott et al. (2013)	Bounceback: a school-based support service for young people experiencing difficulties detrimental to their mental and emotional well-being	Individual; in-person	Teachers referred young people with emotional difficulties/mental health issues, which had the potential to cause a crisis or have a negative effect on emotional well-being	Anticipated stigma; confidentiality and trust
Van de Water et al. (2018a)	Task shifted psychotherapeutic PTSD intervention composed of two treatments: Supportive counselling (SC) and prolonged exposure therapy for adolescents (PE-A)	individual; in person	Trauma-exposed adolescents with chronic (at least 3 months) full PTSD or subthreshold PTSD who were entered into the RCT in the first year (2014) were asked to participate	Anticipated stigma; negative labelling; confidentiality and trust; misconceptions of the service; referral agent; direct solution to a problem
Van de Water et al. (2018b)	as above	as above	as above	Confidentiality and trust; misconceptions of the service

**Table 4:** Mixed Methods Appraisal Tool (MMAT) scores per domain and total score, used to assess methodological quality. Scoring key: Y = Yes, criteria met (1 point); N = No, criteria not met/not possible to assess (0 points); *P* = criteria partially met (0.5 points); blank cell = scoring criteria not applicable

	1. Qualitative design					2. Quantitative (randomised) design					3. Quantitative (non-randomised) design					4. Mixed-methods design					MMAT total Score
Study Identification	1.1	1.2	1.3	1.4	1.5	2.1	2.2	2.3	2.4	2.5	3.1	3.2	3.3	3.4	3.5	4.1	4.2	4.3	4.4	4.5	% (points)
Evans et al. (2015)	Y	Y	Y	Y	Y																100% (5/5)
Fazel (2015)	Y	*P*	Y	Y	Y																90% (4.5/5)
Fazel et al. (2016)	Y	Y	Y	Y	Y																100% (5/5)
Gampetro et al. (2012)	Y	Y	*P*	Y	Y																90% (4.5/5)
Garmy et al. (2015)	Y	Y	Y	Y	Y																100% (5/5)
Gibson & Cartwright (2013)	Y	Y	Y	Y	Y																100% (5/5)
Gibson & Cartwright (2014)	Y	Y	Y	Y	Y																100% (5/5)
Harrison (2019)	Y	*P*	Y	Y	Y																90% (4.5/5)
Harrison (2022)	Y	Y	Y	Y	Y																100% (5/5)
Kendal et al. (2011)	Y	Y	Y	Y	Y																100% (5/5)
Kit et al. (2019)	Y	*P*	Y	Y	Y																90% (4.5/5)
Kvist Lindholm & Zetterqvist Nelson (2014)	Y	Y	Y	Y	Y																100% (5/5)
McKeague et al. (2018)	Y	*P*	Y	Y	Y																90% (4.5/5)
Nabors et al. (1999)	Y	*P*	*P*	*P*	Y																70% (3.5/5)
Nabors et al. (2000)	Y	*P*	*P*	*P*	Y																70% (3.5/5)
Prior (2012a)	Y	Y	Y	Y	Y																100% (5/5)
Prior (2012b)	Y	Y	Y	Y	Y																100% (5/5)
Segrott et al. (2013)	Y	*P*	N	*P*	Y																60% (3/5)
Van de Water et al. (2018a)	Y	*P*	Y	Y	Y																90% (4.5/5)
Van de Water et al. (2018b)	Y	*P*	*P*	Y	Y																80% (4/5)
Pella et al. (2018)						*P*	N	N	N	Y											30%(1.5/5)
Dickinson et al. (2003)	Y	*P*	N	Y	*P*						Y	Y	Y	N	Y	N	N	*P*	N	Y	57% (8.5/15)

(1) Qualitative domain questions: Is the qualitative approach appropriate to answer the research question?; 1.2 Are the qualitative data collection methods adequate to address the research question?; 1.3 Are the findings adequately derived from the data?; 1.4 Is the interpretation of results sufficiently substantiated by data?; 1.5 Is there coherence between qualitative data sources, collection, analysis and interpretation? (2) Quantitative (randomised) domain questions: (2.1) Is randomization appropriately performed? (2.2) Are the groups comparable at baseline? (2.3) Are there complete outcome data? (2.4) Are outcome assessors blinded to the intervention provided? 2.5 Did the participants adhere to the assigned intervention? 3. Quantitative (non-randomised) domain questions: 3.1 Are the participants representative of the target population? 3.2 Are measurements appropriate regarding bout the outcome and the intervention (or exposure)? 3.3 Are there complete outcome data? 3.4 Are the confounders accounted for in the design and analysis? 3.5 During the study period, is the intervention administered (or exposure occurred) as intended? 4. Mixed-methods domain questions: 4.1 Is there an adequate rationale for using a mixed methods design to address the research question?; 4.2 Are the different components of the study effectively integrated to answer the research question?; 4.3 Are the outputs of the integration of qualitative and quantitative components adequately interpreted?; 4.4 Are divergences and inconsistencies between quantitative and qualitative results adequately addressed?; 4.5 Do the difference components of the study adhere to the quality criteria of each tradition of the methods involved?

**Table 5:** Illustrative quotes per theme/subtheme

Key theme and subtheme	Illustrative quotes
a. ***Access-related Factors***	
a.1 Individual agency	“I certainly want to work out what’s best for me without someone else telling me” [participant] (Gibson & Cartwright, 2013) “If I think I need help I’ll go get help” [participant] (Prior, 2012a)
a.2 Referral agent	“It was actually from Mrs Smith, one of the guidance teachers [that I first heard about counselling]” [participant] (Prior, 2012b) “when I joined the high school yeah. I tell my the teacher… I have this problem which can make me not concentrate… and she advised me to see X” [participant] (Fazel et al., 2016)
a.3 Confidentiality and trust	“[the counsellor is] someone you can trust” [participant] (van de Water, et al., 2018) “yes I feel safe because instead of sharing with my friends who might spread it around, I can just talk to online counsellors” [participant] (Kit et al., 2019) “I just feel really trusted with him [counsellor]” [participant] (Segrott et al., 2013)
a.4 Direct solution to a problem	“I don’t talk to somebody about my past. But I knew I needed help” [participant] (Prior, 2012a) “if I didn’t go to the iZ Hero counselling, I would probably still not know how to handle my problems” [participant] (Kit et al., 2019) “I thought Jan could maybe help me with my problem. just help like she’d gie me options on what to dae[do]” [participant] (Prior, 2012a)
a.5 Misconceptions of the service	“I thought I knew something about what to expect but it turned out to be quite different” [participant] (Harrison, 2019) “the clinic is only for people who are physically ill (e.g., bleeding, coughing, etc.), which implies that both participants and clinic employees did not believe PTSD to be an illness that could be effectively treated by medical professionals ” [author] (van de Water et al., 2018)
b. ***Concerns Related to Stigma***	
b.1 Anticipated stigma	“If people found out you were there then some people can be a bit spiteful” [participant] (Prior, 2012a) “yes, there is [a need for a course like DISA], but it is strange that it takes for granted that girls will feel bad” [participant] (Garmy et al., 2015)
b.2 Negative labelling	“Yes, they bring up negative thoughts all the time and everything and then it feels like as if, then apparently I have low self-esteem or something like that” [participant] (Kvist Lindholm & Zetterqvist Nelson, 2015) “People would “[not] talk to them. They make fun of them,” “say, ‘you are crazy’ and ignore them”, or “judge them” [participant] (van de Water et al., 2018) “they would laugh at me; think I am stupid” [participant] (van de Water et al., 2018)
b.3 Navigating through stigma	“I could relate to TRAVELLERS… I hadn’t thought about life’s a journey before the group. I talked to my friends and told them that we talked about things going on in our lives and my friends thought I was lucky. There was no shame and no teasing” [participant] (Dickinson et al., 2003) “… since it was a small group, we wouldn’t feel intimidated to just tell people stuff. It was more confidential in a sense” [participant] (McKeague et al., 2018)
c. ***The School Setting***	
c.1 Available and accessible care	“You know usually like whenever I’m sick, I could come down here and… they help me get better, and I then could go back to class. So that’s convenient you know” [participant] (Gampetro et al., 2012) “well all the other services I did… you know the NHS, and… it was all very clinical and it wasn’t comfortable. I mean [bounceback] made the effort sort of thing; it was little things like, you know, you could sit and you could eat with them… it’s like you go in and they know how to make you feel warm and welcome” [participant] (Segrott et al., 2013) “I think in the school is better” [participant] (Fazel et al., 2016)
c.2 Competing academic schedules	“I think it just took a lot of time. It took a whole school day and for me that’s really a lot of information that I missed and had to catch up on” [participant] (McKeague et al., 2018)

[participant]: utterances by adolescent participants who have accessed and utilised TSMIs, reported in the findings; [field notes]: ethnographic field notes reported in the findings documenting additional observations; [author]: content from author’s results narrative

In terms of intervention characteristics, these were mostly described in general terms as ‘school counselling service’ or ‘school health service’ (*n* = 11; 50%) [18, 42–45, 47, 48, 51, 52, 56, 57]. Two articles (9%) discussed a different version of DISA (‘depression in Swedish adolescents’), a school-based cognitive-behavioural depression prevention program, with one only targeting girls [54], and the other targeting girls and boys between the age of 13–15 years [53]. DISA was universally implemented as a population-wide intervention with targeted efforts to address the needs of specific groups identified as having increased risk of developing depression [60]. Another two articles (9%) discussed the same intervention of a task-shifted psychotherapeutic post-traumatic stress disorder (PTSD) intervention composed of supported counselling (SC) and prolonged exposure therapy for adolescents (PE-A) [49, 50]. The remaining articles (*n* = 7; 32%) described unique interventions summarised in Table 3 [36, 40, 41, 46, 55, 58, 59].

The quality appraisal scores as per the MMAT assessment are presented in Table 4. The methodological quality rating of included qualitative articles (*n* = 20; 100% of qualitative studies) ranged from 60 to 100% suggesting good overall quality. The quality of the quantitative article (*n* = 1; 100% of quantitative studies) scored 30% on the MMAT and the mixed-methods article (*n* = 1; 100% of mixed methods studies) had good overall quality > 50%. For this review, the relevant data used within the mixed-methods article were qualitative; therefore, we conducted a subsequent MMAT rating for the qualitative aspect of this study which also had good methodological quality (60%). Qualitative methodological limitations were associated with a lack of or insufficient details on analysis and data collection procedures and linking these methods back to the research question with reference to relevant data sources. Quantitative methodological limitations were related to insufficient data reporting, specifically on outcome data. And lastly, the mixed-methods methodological limitations were due to the lack of integration between qualitative and quantitative results (i.e., identifying divergences and inconsistencies).

### Thematic synthesis

Addressing the primary review objective (i), three key themes were identified: (a) access-related factors; (b) concerns related to stigma; and (c) the school setting, which were characterised with subthemes discussed herein. These themes describe both barriers and facilitators, as help-seeking processes are variable and can be facilitated or hindered depending on the circumstance. These themes are described below, and corresponding studies are referenced with the main illustrative quotes provided in Table 5.

### (A) Access-related factors

This was the most reported theme and reflects barriers and facilitators related to accessing school-based services. These experiences are described further through the subthemes “individual agency”, “referral agent”, “confidentiality and trust”, “direct solution to a problem”, and “misconceptions of the service”.

*Individual agency.* This theme describes CYP’s self-agency to seek help from TSMS. Some favoured the self-referral route to act on their own awareness of personal need [46]. CYP thus conceptualised themselves as autonomous and self-determining identities empowered to seek help, without assuming a passive illness identity [57]. Several CYP report receiving information about TSMS from other sources – for example, parents and peers – but “emphasised that, in the end, the decision was theirs” [40]. Some went so far as to make a very clear distinction between them and the counsellor, depicting a ‘dominant role’ to themselves and assigning a ‘relatively minor role’ to the counsellor [42]. Participants emphasised the importance of selecting what they, as ‘*clients’* and ‘*entitled consumers’*, wanted from counselling and tailoring aspects of it to fit their particular needs [42, 56]. However, some CYP identify the power imbalance which may prevent their ability to express agency directly to the counsellor [42].

*Referral agent.* A significant proportion of studies discussed some form of referring agent to access the intervention. Most CYP describe teachers as important agents in mediating a referral or support contact with TSMS [50–52]. Yet, a minority thought a self-referral or parents would be better positioned to make the referral than teachers [46, 51]. Some referred to an adult authority in more general terms – parents, a staff member [42, 46, 49, 57]. Fewer discussed peers as a significant referring agent [42]. Interestingly, however, some participants referred to disclosing their own experiences of therapy to peers deemed at risk, in the hope this would encourage others to seek help [49]. For minority groups, the importance of social recognition and impactful peer interactions were acknowledged as important to motivate and instil confidence to seek help [51]. Beyond the initial referral agent, some studies identify a strategy to facilitate adherence and encourage utilisation for the full duration of the program (i.e., incentives). This was specifically reported by males who suggest the ‘need’ to incentivise or reimburse students for participating in the intervention [48].

*Confidentiality and trust.* This theme was largely referred to as confidentiality and trust of the ‘counsellor’ [18, 44, 49, 50, 57, 59]. This was described both as a barrier and a facilitator whereby the counsellor’s other role within the school heightened students’ awareness to the importance of confidentiality [44]. On the other hand, the counsellor mediated this by building trusting relationships with students which facilitated participants’ engagement in and utilisation of TSMS. This was noted to be particularly important in relation to the sociocultural context of Hong Kong, which seemed to heighten initial fears and uncertainty when students first approached counselling [44]. The authors discuss that this may stem from the relatively lower level of trust that exists outside the family within this context, compared to Western cultures. Hence, establishing trust was deemed as crucial to foster relationships and essential for students to disclose information and overcome initial barriers [44]. This extended to some CYP identifying the counsellor as the most important component in the process of accessing and utilising TSMS [50]. CYP seemed to favour an informal and equal relationship with the counsellor and saw them as reliable, non-judgemental and accepting [18, 44, 45, 47–49]. Similarly, CYP liked the online-based intervention and this format may indeed enhance the process of facilitating access to an intervention producing a sense of “psychological safety” with counsellors portrayed as trustworthy, encouraging and a solution provider [59]. Considering most of the interventions were in-person, the counsellors dual role within the school setting left some students feeling confused about the nature of their relationship [44]. This was further depicted as awkward and embarrassing, with some describing a form of barrier indicating that it was difficult to establish an alliance with new therapists due to frequent staff turnover [47]. This subtheme was also conceptualised in relation to the school environment as it was suggested a different location might be beneficial, as privacy and confidentiality might not be fully assured within schools [55]. Also expressed in relation to the importance of creating a ‘safe space’ of trust, where students can attend the intervention without disclosing their reason for leaving class, ensure flexible appointments to not draw attention to absences, and be located in private, non-visible locations to other students [58]. As such, trust with the teacher was identified as a facilitator [49, 51]. And on the whole, lack of trust and unsupportive networks were a barrier to accessing TSMS [49].

*Direct solution to a problem.* This was firstly described as a barrier, with a higher SCARED (Screen for Child Anxiety Related Disorders) total score being significantly associated with a higher number of children’s perceived barriers [36]. However, when previous coping strategies no longer worked, this facilitated CYP to seek help from school-based care [43]. The author described how the progressive worsening of symptoms, whereby they “did not resolve on their own, and distraction techniques were no longer effective”, led students to describe the intervention as “their last remaining hope” [49]. As such, students approached the intervention with pre-existing problems for which they sought care for [59]. Normalising constructions, such as viewing their difficulties as ‘ordinary problems’ in adolescent development and help-seeking as a ‘problem-solving action’, accordingly assisted in managing stigmatisation concerns [57]. The duration of the intervention was thus perceived by some as short-term to meet immediate needs [43].

*Misconceptions of the service.* This theme exists alongside poor mental health literacy as many students were familiar with the counselling service, but “had little or no concept of what counselling was” nor did they consider it as an option for themselves [44]. As such, some described ‘counselling’ as different from what they expected [50]. Lower parental education was also associated with more perceived treatment barriers [36]. Misconceptions were also framed around the concept of who is ‘sick’, implying that physical illness i.e. bleeding or coughing, rather than mental illness, could only be effectively treated by medical professionals [49]. Previous disappointing attempts to seek help from other services can translate to school-based services, forming a barrier to TSMS [43]. These *misconceptions* were also described as a form of ambivalence within the cultural context [44, 49, 59], as some CYP conceptualised this by comparing TSMS to the non-biomedical healing context [49]. While the sociocultural context and minority status appeared to increase sensitivities for some CYP seeking help [44, 51, 52, 55].

### (B) Concerns related to Stigma

This theme captures the prevailing stigma that exists in the school setting, acting as a significant barrier for CYP to seek help. This theme was characterised by the subthemes: “anticipated stigma”, “negative labelling”, and “navigating through stigma”.

*Anticipated stigma.* Anticipated stigma was described as a prominent barrier to accessing TSMS. This appeared to be exacerbated for interventions targeting more severe symptoms “through the already present marginalisation that came from the manifestations of PTSD symptoms” [49]. Likewise, for services targeting minority groups, such as refugees, who may be managing the dual stigma-related burdens associated with both aspects of their identity, with general settling issues (i.e., language problems, asylum issues), and navigating access to and utilisation of TSMS [51]. This is depicted in quantitative findings with a significant effect for minority status (*p* < 0.05) associated with a higher number of children’s perceived barriers to school-based anxiety treatment [36]. Some studies described how CYP negotiated and managed stigma through a socially mediated process by reframing their experiences of accessing and utilising services [56, 57]. This was also described through varied disclosure to friends and family based upon calculated decisions and relational dynamics [49]. Schools also made a conscious effort to construct a positive targeting experience – by framing the intervention as ‘care’ and ‘additional support’, rather than a focus on problematic behaviours – which seemed to avoid potential stigma [40, 41]. Some students suggested some form of advertisement within the school would be a useful mitigating strategy to overcome stigma-related concerns [47, 48].

*Negative labelling.* Individuals who typically face stigma also encounter negative labelling as seen with the DISA intervention when offered to girls only, making them feel negatively targeted, as though the school expected them to have problems that boys did not [53]. These findings were similarly reported when both genders used the intervention, again feeling positioned as having problems they did not perceive themselves to have, and separated a distinction between those who *need* the intervention versus those who do not [54]. Several other articles describe experiencing negative labelling, or the perceived risk of this negative label as a barrier to accessing TSMS [41, 44, 47, 49, 56]. Quantitative findings report a strong association between teacher-rated externalising behaviours and higher perceived children’s barriers [36]. Participants expressed privacy concerns, in particular with not wanting to be seen by peers – or their parents finding out – and risk certain questioning [36, 44, 52]. This raised concerns on judgement and extended to worry how teachers viewed not just CYP, but how this reflected on other family members too [51]. Others resented being teased or “labelled as crazy by peers” due to participating in TSMS [47]. In quantitative findings, over a third of students did not want other children to ask questions (36.9%) or know that they were visiting the counsellor (37.3%) [36]. Several consequences were associated with this negative labelling, such as social exclusion, and some CYP adopted measures to hide or excuse their attendance at the intervention – this extended beyond the school-setting and for parents too [44, 49]. On the other hand, labelling was seen as a coveted process that offered a desirable label of anti-authoritarian attitude within the school context (due to the intervention appearing to target ‘naughty’ students based upon a large number of a discrete friendship group, governed by misbehaviour, being identified) which brought ‘intervention capital’ – serving a powerful commodity to strengthen their position within peer groups [41]. The authors further reported this label may carry iatrogenic effects as the label can only sustain through resistance to the intervention, as engagement risks losing the elevated status [41].

*Navigating through stigma.* Some studies however report on positive experiences where no stigma was attached to participating in the intervention. Students from ‘TRAVELLERS’ describe there was “no shame and no teasing” and key constructs of the programme and metaphors of ‘life as a journey’ were further favoured as they did not “jump to conclusions” [40]. Similarly with ‘DISCOVER’ the group-based format was perceived as beneficial for realising that people shared experiences and reduced feelings of isolation [55]. Some went so far to identify the school-setting as less stigmatising than other service settings being the preferred location for CYP to receive care [52].

### (C) The School setting

Our last theme reflects the influence of the school setting itself in acting as both a barrier and a facilitator in the process of seeking targeted supports within schools. The subthemes include “available and accessible care” and “competing academic schedules”.

*Available and accessible care.* The school setting for mental health care provision was identified as available and accessible [18, 44–46]. Insofar as, some students report they would not have otherwise had the opportunity for nor actively pursued mental health support [45]. Care provided in the school was further characterised as convenient and familiar [52, 55], which led to improve access to other medical care too [18]. The embedded, familiar nature of TSMS therefore provided easier access and more opportunities for students to engage [44]. To the extent that by embedding counsellors within the school setting, the integration of the counselling service with other school activities works to normalise engagement with mental health services [57]. That being said, the school environment was identified as busy and hectic, so “appointments outside of school would probably be calmer” and govern greater privacy with fewer interruptions [48, 52]. This similarly relates to restricted intervention session times and sessions clashing with academic commitments [36, 46–48, 55, 58].

*Competing academic schedules.* A conflict was described between attending intervention sessions and missing lesson time [36, 46–48, 55, 58]. This was a prominent concern for the ‘DISCOVER’ workshop lasting a full day; which was perceived as too time consuming [55]. It was therefore important for CYP to ensure the same class was not missed each week, with some suggesting that teachers and peers provide copies of missed work during intervention sessions to prevent poor academic outcomes [48, 49]. These findings characterised concerns of missing classwork, which was the most commonly endorsed treatment barrier (45.3%) [36].

#### High-income settings/Low-middle-income settings subgroup analysis

For review objective [2], the majority of the included studies (*n* = 15; 94%) were conducted in high-income settings, with only one study from a middle-income setting (*n* = 1; 6%) [49, 50], and no studies from low-income settings. As such, no subgroup analyses between HIS and LMIS were conducted, as a single study does not provide a sufficient, or valid source of information.

## Discussion

This review examined evidence on processes that affect CYP seeking help from TSMS. We have provided a comprehensive synthesis of help-seeking processes reported by CYP in relation to accessing and utilising TSMS. The thematic synthesis thus draws on 16 eligible studies totalling 22 articles of school-based samples and reflecting data reported by CYP themselves.

Similar ideas recurred throughout each article forming several key themes. We found evidence of self-agency and autonomy being important for CYP in the help-seeking process. As reported in previous research, this may reflect the active role CYP adopt during this developmental period to ascertain their agency to seek help for mental health conditions and self-advocate their unique needs [1, 5]. Yet many still alluded to a crucially required referring agent, most often in the form of a significant adult. This is consistent with key CYP help-seeking models that discuss the special feature of CYP help-seeking is that a key adult, generally a parent, plays a central role in the help-seeking process throughout this period [61–64]. Help-seeking was thus constructed as a dichotomous relationship with CYP framed as ‘active agents’ versus ‘passive recipients’ [25, 56]. This may resonate with previous research that identifies how contradictory societal pressures and social norms influence the type of referral – self or other. This may form an ambivalence towards help-seeking for CYP, with a positive attitude for some, and a more negative attitude for others [65]. A facilitator was also described as a strategy to utilise TSMS (i.e., incentives), however, such strategies risk not only affecting the validity of the results, but it is difficult to draw accurate conclusions about the underlying processes associated with service use when utilising these strategies.

Confidentiality and trust were essential to access TSMS, whist also relevant in the process of making optimal use of the service (i.e., through disclosing information to the counsellor). Previous literature reports how ‘conditional disclosure’ can offer a framework for understanding the help-seeking processes of CYP. Their insights into the circumstances under which they feel comfortable disclosing their difficulties could inform strategies to facilitate CYP access to care [66]. As such, a noteworthy amount of the help-seeking process was governed by trust in a formal ‘counsellor’. The importance of both concepts of confidentiality and trust has been emphasised within the broader literature [14, 61, 66–69]. Interestingly, only one intervention was online, which was deemed to produce a sense of “psychological safety” [59]. This finding has been depicted in a scoping review focused on identifying evidence-informed uses of technology for mental health service provision which found young people are more open and confident when online, and anonymity is an important aspect of the technology that engages young people [70]. Despite aligning with previous findings, we cannot draw definitive conclusions on online-based TSMS as only one unique study was included in this review and may therefore not be highly representative or widely applicable.

Stigma-related concerns were pervasive, acting as a form of barrier in the process of seeking care, which is concordant with findings from previous reviews investigating mental health-related stigma [9, 71, 72]. This review provides further evidence that many CYP anticipated stigma develops because of concerns of acquiring a negative label, primarily centred on peers in the school-setting. This resonates with the idea that the school-setting for mental health care can be stigmatising and peer-related concerns govern help-seeking actions [71, 73]. Some indicated stigma-related concerns not only affect the labelled individual, but extend to family members too [51]. Labels can thus be collective and prevail to a group identity which can perpetuate harmful stereotypes and discrimination [72]. Hence, some students distance themselves from potentially needed support due to these stigma-related concerns and to avoid possible stigmatising labels. Some studies identified several mitigating strategies on an individual and structural level that are employed out of stigma-related concerns, which have been extensively researched from the perspective of people with lived experience (PWLE) of mental health conditions and accordingly devised key components of stigma reduction programmes [72]. While other literature provides suggestions from CYP on ways to be discreet and sensitive to vulnerability when it comes to wellbeing support within schools [74]. Even with a comprehensive exploration of help-seeking processes, the findings indicate that stigma is a core concern, persisting prominently even when considering a broad range of influences.

The findings from this review did show promise of navigating through stigma with interventions using non-stigmatising mental health language – a widely disseminated notion to counteract stigma [72, 75]. As such, using empowering language and normalising concepts may avoid undesirable stigmatisation and crucially reject internalised stigma. Similarly, prominent studies have also recommended ways that schools can improve mental health literacy to reduce the barrier of stigma to accessing school mental health services [7, 76]. On a structural level, it is thus essential for schools to employ these strategies to attempt to promote help-seeking behaviours. One study identified the school-setting as less stigmatising than other locations, being CYP preferred location to receive care [52]. This finding offers a positive and auspicious outlook on TSMS which may indeed resonate with previous findings that the familiarity of schools may make treatment more acceptable [77]. Further, a group-based format seemed to be favoured leading to enhance peer support and potentially extending beyond the intervention itself and counteract stigmatising beliefs in the school more generally. This was even described with some CYP disclosing their own experiences of utilising TSMS to encourage others to seek help. This shows potential for ways to increase help-seeking through collective shared experience and peer relations [49, 78], and recognises the importance of PWLE as agents to counteract stigma [72].

In general, TSMS were considered available and accessible and facilitated pathways to other services of care. In particular, CYP favoured informality when utilising TSMS which is consistent with the broader literature [63, 79]. However, considerable concerns were raised in many articles on competing academic schedules within schools. These concerns are reflected in previous research identifying the importance of receiving buy-in from all school personnel, whilst also involving them in the co-design of interventions which may minimise disruption to school routine, and to further enhance their sustainability [28, 80]. Recent research extends on these ideas identifying the importance of including CYP themselves in the decision making for wellbeing provision within schools to achieve a desired integrated systems approach [74].

This review found most CYP sought help once they fully exhausted all methods to manage their symptoms themselves. The literature reports similar findings that CYP preference to self-manage their symptoms was a significant barrier to seeking help for mental health [66, 81–83]. Another focal barrier was based upon misconceptions of TSMS, based upon poor mental health literacy. These barriers are widely discussed in literature that impact CYP engaging with TSMS [10, 14, 84].

Ambivalence was also discussed in relation to cultural values; TSMS may therefore require a culturally sensitive approach and in some settings may even require collaboration with community stakeholders to successfully implement interventions in schools. Interestingly, several studies specified the school setting within HIS in low-resource areas and delineated how barriers may be exacerbated in these settings. Therefore, the school setting may be the only place where underserved and hard-to-reach CYP receive care [18, 55]. Despite being unable to contrast findings from different settings, these findings reiterate previous research that help-seeking is not homogenous and help-avoidance behaviours are more acute among certain groups (i.e., low socioeconomic, minority ethnic), which perpetuates inequities for accessing mental health support [49, 85]. Consequently, it is crucial school-based services are flexible to the student’s needs, especially in ethnically and economically diverse contexts. The reported findings are enduring, far-reaching and underscore the inherent need for widescale education on mental health, services available, and treatment.

### Strengths and limitations

To the best of our knowledge, this is the first review to synthesise all available data on help-seeking processes related to TSMS, based upon evidence coming from CYP who engaged with and utilised TSMS. Prior studies not only derive from hypothetical scenarios [65], but frequently report from another informant – namely the provider or parent – rather than CYP. As such, the lived experience of CYP provides enriched data uncovering authentic experiences on the processes that affect accessing and utilising TSMS. However, the findings from this review must be interpreted with specific limitations. Firstly, despite employing a thorough search strategy, screening several databases, and carrying out extensive manual methods for citation tracking and expert consultations, we cannot guarantee that we have captured all the relevant articles for inclusion. This should be considered in conjunction with the narrow inclusion and exclusion criteria whereby only published, peer-reviewed journal articles were eligible for inclusion. Nonetheless, it seems implausible that publication status would generate substantial bias for the topic of enquiry. Another limitation is the absence of a quantitative assessment of inter-rater reliability; however, consistent screening was ensured through discussions and agreement among raters. Most included studies were from HIS, so it was impractical to contrast subgroup differences based on evidence from HIS and LMIS. This further warrants a limitation on the scope of this review restricting the generalisability and applicability of the findings to other contexts. There should, however, be caution in considering the lack of potential differences between settings. It may also still be argued that the focus on low-resource areas in HIS in some of the included articles increases the representativeness of the population of interest, due to capturing various settings in high-income contexts. It should be noted that only one quantitative study was included, thus the review consists of predominantly qualitative data which posed hindrance to conducting a narrative synthesis as we set out to do. This further affected the representativeness of the perspectives of CYP for whom this review reflects, with the relatively small, aggregated sample of CYP included in this review. Lastly, not all CYP are enrolled in schools, even in some HIS [86]. Hence, CYP facing significant health inequities may not be represented in studies conducted in schools.

### Implications

*Research.* With the limited quantitative studies included in this review, researchers should prioritise efforts to increase quantitative research to benefit from improved generalisability and a balanced evidence-base to inform decision-making. An integrated approach with quantitative and qualitative data may be more pervasive and carry substantial importance in the process of decision-making in policy and practice for TSMS. It may also seem plausible that going forward much support for young people might be offered online, so it is important to carry out further research to understand more about influences for accessing and utilising that form of support. Lastly, researchers should prioritise conducting more research in LMIS, as they are currently significantly under-represented in studies investigating help-seeking processes related to TSMS.

*Clinical practice in schools.* The findings of this review should inform future intervention design for TSMS in line with the processes that facilitate intervention participation. Most significantly this should be centred on promoting mental health literacy and mitigating the risk of stigma and negative labelling that pertain CYP help-seeking in the school. As most of these stigma-related concerns are seen in conjunction with peer-related concerns, schools must ensure they have a referral pathway that is deemed private and confidential. Namely, schools should support self-referral pathways and ensure appropriate adult referring agents are linked to school-based services that are informal and trustworthy – critical in the help-seeking process for CYP [63].

## Conclusion

The growth of TSMS to broaden pathways for CYP to receive essential mental health support underscores the importance of understanding the processes of engagement with these services from the perspective of CYP themselves. This systematic review of qualitative, quantitative and mixed-methods evidence uncovers the dynamic interplay between various factors that contribute to the help-seeking process of accessing and engaging with TSMS, an important contribution to the literature yet to be integrated. This should guide the delivery and development of TSMS to facilitate access to this kind of support, promote help-seeking behaviours, and lastly, the gaps identified should direct researchers to investigate TSMS in LMIS and prioritise increasing quantitative studies.

### Electronic supplementary material

Below is the link to the electronic supplementary material.

Supplementary Material 1

## Data Availability

All data generated or analysed during this study are included in this published article and its supplementary information files.

## Abbreviations

TSMS Targeted School: based Mental Health Services
WHO: World Health Organisation
DALYS: Disability-adjusted life years
HIS: High-income settings
LMIS: Low-and-middle- income settings
PRISMA: Preferred Reporting Items for Systematic Reviews and Meta-Analyses
EMBASE: Excerpta Medica dataBASE
MEDLINE: Medical Literature Analysis and Retrieval System Online
CINAHL: Cumulated Index to Nursing and Allied Health Literature
ERIC: Electronic Registration Information Centre
MMAT: Mixed-methods appraisal tool
DISA: ‘Depression in Swedish adolescents’
PTSD: Post traumatic stress disorder
SC: Supported Counselling
PE-A: Prolonged exposure therapy for adolescents
SCARED: Screen for Child Anxiety Related Disorders
PWLE: People with lived experience
CYP: Children and young people
