# Supplementary material for: Help-seeking processes related to targeted school-based mental health services: systematic review
Source: BMC Public Health. 2024 May 2;24:1217. doi: 10.1186/s12889-024-18714-4 (PMC11065683; doi:10.1186/s12889-024-18714-4)
Supplement: Supplementary file 1 — Supplementary Material 1 [file 12889_2024_18714_MOESM1_ESM.docx]

**Supplement 1: PRISMA 2020 checklist**

| **Section and Topic** | **Item #** | **Checklist item** | **Location where item is reported** |
| --- | --- | --- | --- |
| **TITLE** | | |  |
| Title | 1 | Identify the report as a systematic review. | Title page |
| **ABSTRACT** | | |  |
| Abstract | 2 | See the PRISMA 2020 for Abstracts checklist. | Page 2 |
| **INTRODUCTION** | | |  |
| Rationale | 3 | Describe the rationale for the review in the context of existing knowledge. | Page 4-6 |
| Objectives | 4 | Provide an explicit statement of the objective(s) or question(s) the review addresses. | Page 6-7 |
| **METHODS** | | |  |
| Eligibility criteria | 5 | Specify the inclusion and exclusion criteria for the review and how studies were grouped for the syntheses. | Page 7-8 |
| Information sources | 6 | Specify all databases, registers, websites, organisations, reference lists and other sources searched or consulted to identify studies. Specify the date when each source was last searched or consulted. | Page 7-8 |
| Search strategy | 7 | Present the full search strategies for all databases, registers and websites, including any filters and limits used. | Supplement 2 |
| Selection process | 8 | Specify the methods used to decide whether a study met the inclusion criteria of the review, including how many reviewers screened each record and each report retrieved, whether they worked independently, and if applicable, details of automation tools used in the process. | Page 8-9 |
| Data collection process | 9 | Specify the methods used to collect data from reports, including how many reviewers collected data from each report, whether they worked independently, any processes for obtaining or confirming data from study investigators, and if applicable, details of automation tools used in the process. | Page 9 |
| Data items | 10a | List and define all outcomes for which data were sought. Specify whether all results that were compatible with each outcome domain in each study were sought (e.g. for all measures, time points, analyses), and if not, the methods used to decide which results to collect. | n/a |
|  | 10b | List and define all other variables for which data were sought (e.g. participant and intervention characteristics, funding sources). Describe any assumptions made about any missing or unclear information. | Page 9 |
| Study risk of bias assessment | 11 | Specify the methods used to assess risk of bias in the included studies, including details of the tool(s) used, how many reviewers assessed each study and whether they worked independently, and if applicable, details of automation tools used in the process. | Page 10 |
| Effect measures | 12 | Specify for each outcome the effect measure(s) (e.g. risk ratio, mean difference) used in the synthesis or presentation of results. | n/a |
| Synthesis methods | 13a | Describe the processes used to decide which studies were eligible for each synthesis (e.g. tabulating the study intervention characteristics and comparing against the planned groups for each synthesis (item #5)). | Page 10-11 |
|  | 13b | Describe any methods required to prepare the data for presentation or synthesis, such as handling of missing summary statistics, or data conversions. | Page 10-11 |
|  | 13c | Describe any methods used to tabulate or visually display results of individual studies and syntheses. | Page 10-11 |
|  | 13d | Describe any methods used to synthesize results and provide a rationale for the choice(s). If meta-analysis was performed, describe the model(s), method(s) to identify the presence and extent of statistical heterogeneity, and software package(s) used. | Page 10-11 |
|  | 13e | Describe any methods used to explore possible causes of heterogeneity among study results (e.g. subgroup analysis, meta-regression). | Page 10-11 |
|  | 13f | Describe any sensitivity analyses conducted to assess robustness of the synthesized results. | n/a |
| Reporting bias assessment | 14 | Describe any methods used to assess risk of bias due to missing results in a synthesis (arising from reporting biases). | n/a |
| Certainty assessment | 15 | Describe any methods used to assess certainty (or confidence) in the body of evidence for an outcome. | n/a |
| **RESULTS** | | |  |
| Study selection | 16a | Describe the results of the search and selection process, from the number of records identified in the search to the number of studies included in the review, ideally using a flow diagram. | Page 11 |
|  | 16b | Cite studies that might appear to meet the inclusion criteria, but which were excluded, and explain why they were excluded. | PRISMA Diagram |
| Study characteristics | 17 | Cite each included study and present its characteristics. | Page 11-13 |
| Risk of bias in studies | 18 | Present assessments of risk of bias for each included study. | Page 13 |
| Results of individual studies | 19 | For all outcomes, present, for each study: (a) summary statistics for each group (where appropriate) and (b) an effect estimate and its precision (e.g. confidence/credible interval), ideally using structured tables or plots. | n/a |
| Results of syntheses | 20a | For each synthesis, briefly summarise the characteristics and risk of bias among contributing studies. | Page 13-20 |
|  | 20b | Present results of all statistical syntheses conducted. If meta-analysis was done, present for each the summary estimate and its precision (e.g. confidence/credible interval) and measures of statistical heterogeneity. If comparing groups, describe the direction of the effect. | n/a |
|  | 20c | Present results of all investigations of possible causes of heterogeneity among study results. | n/a |
|  | 20d | Present results of all sensitivity analyses conducted to assess the robustness of the synthesized results. | n/a |
| Reporting biases | 21 | Present assessments of risk of bias due to missing results (arising from reporting biases) for each synthesis assessed. | n/a |
| Certainty of evidence | 22 | Present assessments of certainty (or confidence) in the body of evidence for each outcome assessed. | n/a |
| **DISCUSSION** | | |  |
| Discussion | 23a | Provide a general interpretation of the results in the context of other evidence. | Page 20-24 |
|  | 23b | Discuss any limitations of the evidence included in the review. | Page 24-25 |
|  | 23c | Discuss any limitations of the review processes used. | Page 24-25 |
|  | 23d | Discuss implications of the results for practice, policy, and future research. | Page 25-26 |
| **OTHER INFORMATION** | | |  |
| Registration and protocol | 24a | Provide registration information for the review, including register name and registration number, or state that the review was not registered. | Page 3 |
|  | 24b | Indicate where the review protocol can be accessed, or state that a protocol was not prepared. | Page 3 |
|  | 24c | Describe and explain any amendments to information provided at registration or in the protocol. | n/a |
| Support | 25 | Describe sources of financial or non-financial support for the review, and the role of the funders or sponsors in the review. | Page 28 |
| Competing interests | 26 | Declare any competing interests of review authors. | Page 28 |
| Availability of data, code and other materials | 27 | Report which of the following are publicly available and where they can be found: template data collection forms; data extracted from included studies; data used for all analyses; analytic code; any other materials used in the review. | Page 28 |

Page MJ, McKenzie JE, Bossuyt PM, Boutron I, Hoffmann TC, Mulrow CD, et al. The PRISMA 2020 statement: an updated guideline for reporting systematic reviews. BMJ 2021;372:n71. doi: 10.1136/bmj.n71

**Supplement 2: Full search strategy**

Embase <1974 to 2023 March 21>

1  ((treatment or care) adj3 (seek* or sought or request*)).mp. 59298

2  ((help or assistance) adj3 (seek* or sought or request*)).mp. 34453

3  ((treatment or care or help or assistance) adj3 (facilitate* or enhance* or enable* or opportunit* or encourage* or motivate* or motivating or promote* or promoting or influence* or influencing or barrier* or challenge* or challenging or constrain* or constrict* or deter* or difficult* or discourag* or disincentive* or encumber* or encumbrance or hinder* or hindrance* or impair* or impede* or limit* or delay or obstruct* or problem or perception* or perspective* or view* or experience* or need* or attitude* or opinion* or quality or implementat* or uptake* or utilis* or utiliz* or use* or accept* or availab* or access* or reciev*)).mp. 1615376

4  exp patient attitude/ or exp help seeking behaviour/ 502711

5  1 or 2 or 3 or 42057108

6  (adolescen* or youth or teen* or teenager* or ((student* or pupil*) adj2 school) or children or boy* or girl*).mp.3151152

7  ("secondary education" or "middle education").mp. 5423

8  ("secondary school*" or "middle school*" or "high school" or "junior high school*" or "senior school*" or "public school*" or "private school*").mp. 84193
9 exp high school/ or exp middle school/ or exp school child/ or exp high school student/ or exp middle school student/ 456454

10  adolescent/ or exp boy/ or exp girl/ 1789494

11  6 or 7 or 8 or 9 or 10 3260771

12  ("anxiety disorder*" or anxiet* or depress* or "depressive disorder*" or self-harm or self-injury or suicid* or well?being or resilien or "mood disorder*" or "attention deficit disorder*" or "attention hyperactivity disorder*" or ADHD or "neurodevelopment disorder*" or schizophrenia or psychos* or "psychotic disorder*" or "schizoaffective disorder*" or "schizophreniform disorder*" or "psychotic affective disorder*" or "psychotic mood disorder" or "affective psychosis" or "bipolar disorder*" or "bipolar affective psychosis" or "bipolar affective disorder*" or bipolar or "post?traumatic stress disorder*" or "traumatic stress disorder*" or "stress disorder*" or "acute stress reaction" or "panic disorder*" or OCD or "obsessive compulsive disorder*").mp. 1904750

13 (mental* adj3 (health or illness* or condition* or disabilit* or disorder* or disease* or impair* or problem* or stress or wellbeing or "well being")).mp. 754914

14 (psychiatri* adj3 (health or illness* or condition* or disabilit* or disorder* or disease* or impair* or problem* or stress or wellbeing or "well being")).mp. 129396

15 (psychologic* adj3 (health or illness* or condition* or disabilit* or disorder* or disease* or impair* or problem* or stress or wellbeing or "well being")).mp. 107361

16 mental deterioration/ or mental stress/ or exp community mental health/ or exp mental disease/ or mental instability/ or mental capacity/ or exp mental health/ or exp emotional stability/ or emotional well-being/ or emotional disorder/ or emotional stress/ 2791489

17 12 or 13 or 14 or 15 or 16 3556149

18 (targeted adj3 (service* or program* or therap* or initiative* or scheme* or intervention* or treatment* or prevention*)).mp. 228341
19 (selective adj3 (service* or program* or therap* or initiative* or scheme* or intervention* or treatment* or prevention*)).mp. 19308
20 (indicated adj3 (service* or program* or therap* or initiative* or scheme* or intervention* or treatment* or prevention*)).mp. 47423

21 18 or 19 or 20 292892

22 (school* or education*).mp. 2178007

23 21 and 22 19668

24 ((school* or education*) and ("mental health service*" or "mental healthcare" or "mental health care" or "mental care" or "psychiatric service*" or "psychiatric health care" or "psychiatric healthcare" or "psychiatric care" or "psychiatric service*")).mp. 21911

25 exp school health service/ 20904

26 23 or 24 or 25 60960

27 5 and 11 and 17 and 26 4043
